# Prevention and Control Strategies to Counter Dengue Virus Infection

**DOI:** 10.3389/fcimb.2017.00336

**Published:** 2017-07-25

**Authors:** Irfan A. Rather, Hilal A. Parray, Jameel B. Lone, Woon K. Paek, Jeongheui Lim, Vivek K. Bajpai, Yong-Ha Park

**Affiliations:** ^1^Department of Applied Microbiology and Biotechnology, School of Biotechnology, Yeungnam University Gyeongsan, South Korea; ^2^Department of Biotechnology, Daegu University Gyungsan, South Korea; ^3^National Science Museum, Ministry of Science, ICT and Future Planning Daejeon, South Korea

**Keywords:** dengue virus, infection, vaccine, disease, fever

## Abstract

Dengue is currently the highest and rapidly spreading vector-borne viral disease, which can lead to mortality in its severe form. The globally endemic dengue poses as a public health and economic challenge that has been attempted to suppress though application of various prevention and control techniques. Therefore, broad spectrum techniques, that are efficient, cost-effective, and environmentally sustainable, are proposed and practiced in dengue-endemic regions. The development of vaccines and immunotherapies have introduced a new dimension for effective dengue control and prevention. Thus, the present study focuses on the preventive and control strategies that are currently employed to counter dengue. While traditional control strategies bring temporary sustainability alone, implementation of novel biotechnological interventions, such as sterile insect technique, paratransgenesis, and production of genetically modified vectors, has improved the efficacy of the traditional strategies. Although a large-scale vector control strategy can be limited, innovative vaccine candidates have provided evidence for promising dengue prevention measures. The use of tetravalent dengue vaccine (CYD-TDV) has been the most effective so far in treating dengue infections. Nonetheless, challenges and limitation hinder the progress of developing integrated intervention methods and vaccines; while the improvement in the latest techniques and vaccine formulation continues, one can hope for a future without the threat of dengue virus.

## Introduction

Dengue is a mosquito-borne viral infection (Simmons et al., 2012), which has affected almost 2.5 billion people around the globe (Koh et al., 2008). It is transmitted by vector species *Aedes aegypti* and poses a global threat to humans due to its high adaptability to urban communities (Araújo et al., 2015). In 2012, WHO reported that dengue outbreaks place a large burden on communities, healthcare systems, and economies in most tropical countries worldwide. According to WHO, Asia, Americas, Africa, and the Mediterranean regions are affected by the emerging and prevailing DENV (WHO, 2012).

**Figure 1:**
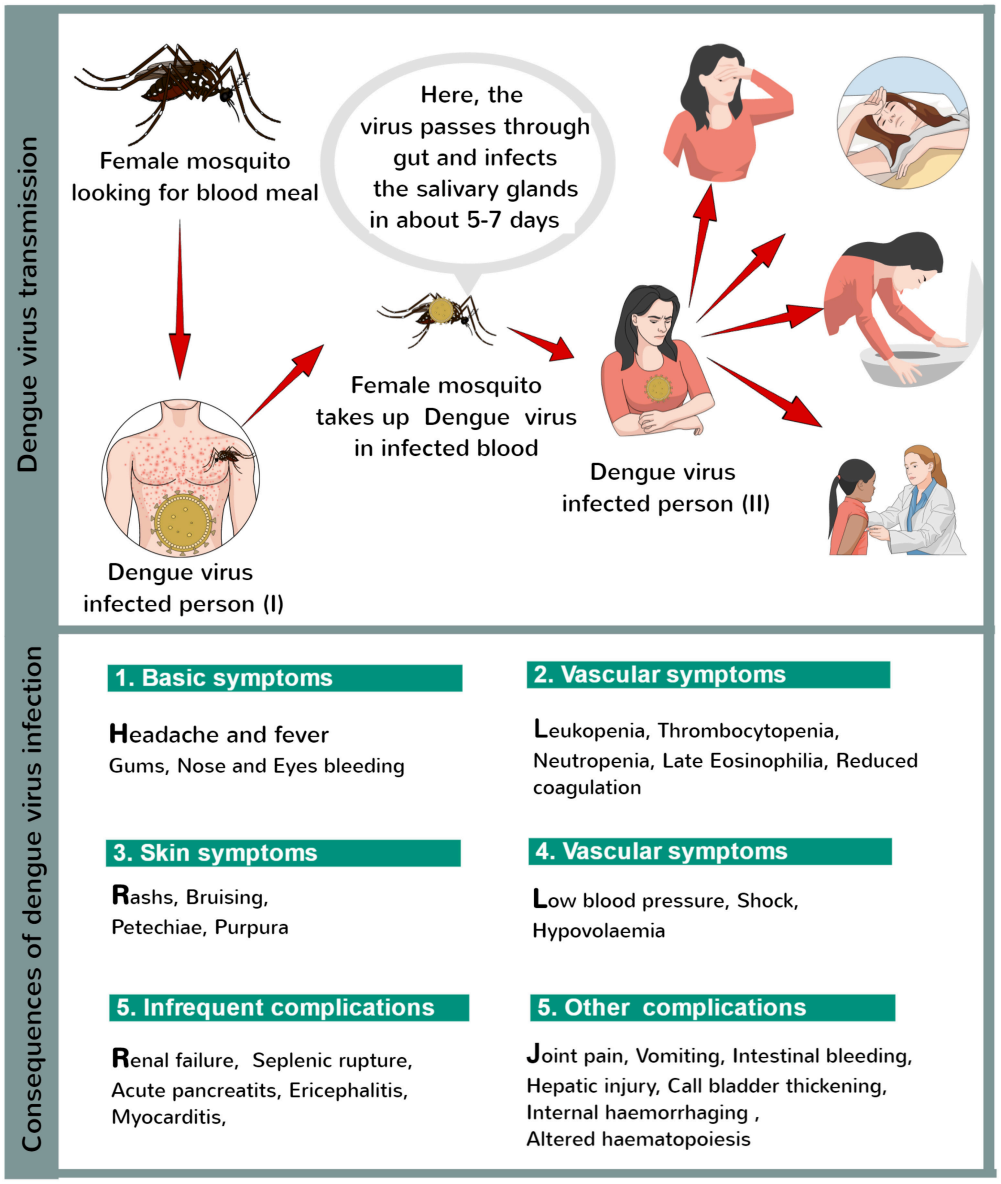
Dengue virus infection.

Recently, Bhatt et al., estimated about 390 million DENV infections occurring each year, of which 96 million were seemingly evident (Bhatt et al., 2013). The DENV infection starts with mild fever, and further leads to many other consequences (Figure 1). However, preventive strategies for DENV have been developed in the form of vector control, including chemical, biological, and physical controls. Apart from general control strategies, development of vaccines have offered effective prevention and control of this disease (DeRoeck et al., 2003).

Similarly, the following study aims to discuss the prevention and control strategies to counter DENV, including the development of immunotherapies and vaccines. It also examines the challenges confronted in the effective implementation of these strategies in light of peer reviewed literature, and draws a conclusion of the research.

### Prevention and control strategies

The prevention and control methods are divided into three categories, which have been discussed accordingly.

### Physical control

#### GIS mapping of dengue foci

Among the advanced techniques used for location of DENV, GIS mapping has been efficient in locating dengue concentrations. By locating dengue seri-positive cases within the study area, dengue transmission can be prevented by locating dengue foci, and then treating them with diverse preventive strategies (Gandhi et al., 2017). Kittayapong et al., showed that GIS mapping not only allowed better surveillance and community-based intervention programs for suppressing dengue; it also determined the rate of successful control in the mapped areas. In their study, surveillance of the mapped dengue foci determined the major breeding sites of *A. egypti* mosquitoes to be water containers and bath basins (Kittayapong et al., 2008).

#### Focused and effective surveillance

Surveillance provides fundamental information on the assessment of risk, outbreak reaction, program evaluation and guidance, as well as delivers timely responses to prevent and control dengue (Wilder-Smith et al., 2012). Surveillance enables the understanding of spatiotemporal distribution of dengue cases, and provides entomological and epidemiological links for better planning (WHO, 2012; Scarpino et al., 2017). On the other hand, these programs are not focused on the elimination of dengue vector (Abbas et al., 2014). The eruption of dengue in Singapore, after decades of surveillance, indicated unsustainable vector control measures and ineffective surveillance (Ooi et al., 2006) in 2005 (Koh et al., 2008). An effective surveillance system aiming at vector identification (Gómez-Dantés and Willoquet, 2009) and eradication (Abbas et al., 2014), providing the underlying information regarding vector concentration and its breeding, will prove beneficial in controlling vector species.

#### Determination of oviposition sites

As determined by Morrison et al., *Aedes aegypti* females lay eggs above the water in containers or jars and so on for their survival improvement (Morrison et al., 2004). To detect and reduce the population density of dengue vectors, it is necessary to determine the behavioral pattern of vectors. Wong et al., studied the oviposition pattern of *A. aegypti* and reported that strong intra-specie affinity may be an indication of targeting vector specie. Moreover, once the oviposition sites have been determined, introduction of strategies that eliminate mosquito population at a later developmental stage will increase the efficacy of control strategies (Wong et al., 2011). Recently, introducing oviposition-based innovative techniques have shown promising results in intensifying control of vector species (Johnson et al., 2017).

#### Community-based control programs

Community-based control programs are developed with the aim to educate the community about the measures for the extermination of mosquito breeding sites. People in a community are divided into various groups depending upon their level of education and understanding (Abbas et al., 2014). The significance of community-based programs for elimination of dengue mosquitoes in Kerala district (George et al., 2017), Mexico (Tapia-Conyer et al., 2012), and Cuba (Vanlerberghe et al., 2010) has been proven in the form of elevated awareness among the communities. Through community involvement, a variety of techniques can be integrated for maximum control of vector population (Heintze et al., 2007; Pérez-Guerra et al., 2009; Shriram et al., 2009), such as, the combination of community-based program and chemical control of *A. aegypti* have yielded significant results in Cuba (Baly et al., 2007).

#### Education of prevention strategies

It has been noted that the success of community-based strategies depends upon the knowledge, education, and behavior of the people, and strategies involved (Nam et al., 2005). Education serves as a basis for an ability of an individual to identify and deal with vector habitats, and use preventive measures. Madeira et al., emphasized that distribution of information brings awareness in order to control dengue, and provides necessary measures for the destruction of vector habitats (Madeira et al., 2002). A recent study in Thailand showed that education of prevention strategies through media also played a vital role in developing awareness (Boonchutima et al., 2017).

### Biological control

#### Paratransgenesis and use of wolbachia

Nowadays, genetic control of *A. aegypti* has risen as a set of promising techniques, among which paratransgenesis is the popular method (Araújo et al., 2015; Ogaugwu and Durvasula, 2017). This approach utilizes genetically-modified symbiotic bacteria that are reintroduced in the vector to colonize the vector population, hence limiting the transmission of disease (Araújo et al., 2015; Wilke and Marrelli, 2015). These genetically modified bacteria cause harmful effects in the host body, dysregulate its sexual cycle, decrease the host competence and interfere with the developmental processes of the vector species, thereby suppressing the vector population (Wilke and Marrelli, 2015). As reported in the study by Jeffery et al., the most effective bacterial agents used is *Wolbachia* (Jeffery et al., 2009; Saldaña et al., 2017), which is a reproductive parasite interfering with the cellular and reproductive mechanisms of the vector species (Araújo et al., 2015; Kamtchum-Tatuene et al., 2016).

#### Vector specie genetic modification

The genetic methods for the control of *A. aegypti* aim at suppressing the population and its replacement or transformation. Therefore, the aim is designed to provide an alternate that could be accounted for providing an effector gene for reduction and inhibition of disease transmission (Reis-Castro, 2012; Carvalho et al., 2014; Jupatanakul et al., 2017). The field release of genetically modified mosquito species in Brazil showed an 85% decline in *A. aegypti* population (Pan American Health Organization, 2014), indicating that genetically modified vector species are innovative and feasible methods used for blocking the transmission of mosquito-borne diseases (Fraser, 2012; Favia, 2015).

#### Use of sterile insect technique (SIT)

As the name indicates, SIT refers to the release of laboratory-sterilized male vectors in the target population. Once released, these male mosquitoes help in suppressing the fecundity rate in female mosquitoes and, consequently control the vector density in urban environments (Dumont and Chiroleu, 2010; Yakob et al., 2017) and transmission of vector-borne diseases (Alphey et al., 2010). According to Oliva et al., SIT is a promising strategy that helps in prevention and control of mosquito-borne diseases. After examining the irradiation effect on sterile male, they stated that sterile males were potential competitors and can help suppress the number of offsprings (Oliva et al., 2012).

#### Use of larvivorous fish and crustacean

Since the larvae of dengue vectors reside in open water bodies, use of larvivorous fish, such as *Poecilia reticulate* (Seng et al., 2008) and *Mesocyclops formosanus* (Kalimuthu et al., 2017) comes as a cost-effective, eco-friendly, and innovate strategy in controlling the population of *A. aegypti* (Abbas et al., 2014; Han et al., 2015; Warbanski et al., 2017). A successful study in Cambodia was carried out to evaluate the efficacy of introducing larvivorous guppy fish (*Poecilia reticulata*) into heavily infested water containers. It showed that the guppy fish in test houses reduced vector larval population by 79% as compared to control houses, thus indicating successful implementation of this strategy (Seng et al., 2008).

### Chemical control

#### Use of insecticides and plant derivatives

The chemical compounds, called insecticides, have been utilized for mosquito control for many decades. These insecticides became the most commonly used integrated strategy; nevertheless, the continuous use developed resistance in the target vector population, and can induce negative impacts on the environment (Araújo et al., 2015). To counter the effects of these compounds, researchers developed alternative control method i.e., introduction of plant-based insecticides that can sustain and induce less toxicity in environment than synthetic insecticides (Ghosh et al., 2012). These plant-based insecticides can be developed from different plant parts (leaves, stem, roots) and/or herbal extracts, such as, *Cipadessa baccifera* (Ramkumar et al., 2015), *Callistemon rigidus* (Pierre et al., 2014), *Erythrina indica, and Asparagus racemosus* (Govindarajan and Sivakumar, 2015). Furthermore, these plant derivatives are not only limited to produce insecticides; however, they have also proved their efficiency as potential repellents against *A. aegypti* (Araújo et al., 2015; Govindarajan and Sivakumar, 2015).

#### Use of insect growth regulators (IGRs)

Among other known chemical compounds, insect growth regulators (IGRs) are used for hindering the growth and development in insects. During early stages of development, IGRs induce changes that kills the insect before becoming an adult. There are number of IGRs such as, diflubenzuron, endotoxins, and methoprene that have been used to counter viral infections spread by *A. aegypti* (Abbas et al., 2014). According to Lau et al., field population of vector species develops resistance to certain IGRs; and in their study, they found that cyromazine showed effective results in attenuating larval population indices of *A. aegypti* (Lau et al., 2015).

#### Use of pheromones as “attract-and-kill” approach

The practical application of pheromones as a part of integrated pest management (IPM) has been well-documented in various fields. In a recent integrated approach using pheromones, also termed attracticides, and IGRs, Nagpal et al., demonstrated the prevention of developmental stages from eggs to adults (Nagpal et al., 2015). In this study, larvae in test containers were found in a greater number than controls containers, which indicated that using attracticides hampers the progression of adulthood in *A. aegypti* and is effective in field conditions (Nagpal et al., 2015). Another study developed an uncomplicated “lethal lure control” based on attract-and-kill strategy and found that the pheromone (caproic acid)-insecticide (temephos) combination not only attracted mosquitoes, but also restricted hatching of eggs and killed the larvae, thus elaborating its significance (Ong and Jaal, 2015).

### Development of immunotherapies and vaccines

Although no specific vaccine for dengue has been licensed at commercial scale, several candidates have been undergoing a developmental phase. Some of these are discussed below:

#### Live, attenuated dengue vaccines

Among the vaccines having been improved, the development of live, attenuated vaccines, known as ChemariVax-Dengue (CYD)-based bivalent and tetravalent vaccines (CYD-TDV), have shown protection against DENV in a trial conducted in Mexico (Dayan et al., 2014). The study determined that the group receiving bivalent vaccine showed an immune response against CYD serotype 3, while the immune response rates of other group receiving first injection of CYD-TDV were generally higher and well-tolerated (Dayan et al., 2014). Evidences from randomized, controlled studies have emphasized the importance of tetravalent CYD vaccine in Asian (Capeding et al., 2014), Thai (Sabchareon et al., 2012), and Latin American (Villar et al., 2015) children along with adults in Singapore (Sin Leo et al., 2012), suggesting its potential in providing protection against various CYD serotypes. However, studies also suggested the lower risk of CYD in CYD-TDV vaccinated children aged 2–16 years than the unvaccinated control group (Hadinegoro et al., 2015), neutralization of antibody response to dengue serotypes, and safe profile of CYD-TDV (Qiao et al., 2011; Villar et al., 2013). The available candidates for dengue vaccine are listed in Table 1.

**Table 1:** Current Vaccine Candidates for Dengue Prevention (Source:Sandrasegaran, 2016).

**Vaccine type**	**Developer**	**Process**	**Progress**
Live, attenuated chimeric (recombinant)	Acambis/Sanofi Pasteur	Insertion of genes coding for DENV structural proteins into a yellow fever virus (17D) backbone.	Phase III tetravalent—leading candidate
	Centre for Disease Control (CDC)/Inviragen	Insertion of serotype genes into serotype II (DENV2- PDK53) DNA backbone.	Phase II monovalent
	National Institutes of Health (NIH)/University of Maryland	Insertion of serotype II and III genes into safer, more immunogenic serotype I and IV DNA backbone. Live attenuated DENV Delta-30 mutation	Phase I tetravalent
Live, traditionally attenuated	Walter-Reed Army Institute of Research (WRAIR)/GlaxoSmithKline (GSK)	Attenuation achieved by growing the virus in cultured cells and selecting strains	Phase II tetravalent; technical issues
	Mahidol Institute/Sanofi Pasteur		Phase II tetravalent
Inactivated	GSK	Viruses cultured and killed	Phase I tetravalent
Subunit	Hawaii Biotech	Viral immunogenic envelope is combined with viral non-structural protein antigens to produce recombinant 80% E subunit vaccine	Phase I tetravalent
DNA	WRAIR	Dengue prM-E DNA vaccine incorporating membrane and envelope genes into a plasmid vector	Phase I monovalent

Besides live and/or attenuated vaccines, inactivated and non-replicating vaccines have also been used. The developing non-replicating vaccine approaches focus on recombinant DENV antigens, inactivated viruses, and use of non-replicating transmission agents produced specifically to extract DENV antigens *in vivo*. Using inactivated vaccines also reduce the risk of infection by conferring resistance. Also, subunit vaccines and genetic vaccination have been developed to respond to the inactivated viruses (Swaminathan and Khanna, 2010).

Recent outbreak of Zika virus (ZIKV) epidemics has raised a growing concern in many parts of the world. However, several viral diseases have been controlled using vaccination strategies. Nevertheless, for majority of arthropod transmitted viral diseases, there is no specific vaccine yet. Therefore, exploring potential transmission blocking vaccines (TBV) could halt the viral infection to humans, and could be applied to most of the arboviruses, including chikungunya, DENV, and ZIKV.

#### Development of dengue human infection model

In order to develop the understanding of DENV pathogenesis and effective dengue countermeasures, the evolution of the dengue human infection model (DHIM) is also deemed necessary. Developing a DHIM requires a thorough examination of measures to reduce risks to participants and guidelines for clinical management. Moreover, DHIM serves as a promising research tool, which enables the understanding of pathways for vaccine development, examines the immunological pathogenesis, exploits protection by immune associates, supports the evolution of vaccine clinical development, and would put into effect the efforts for the development of effective vaccines (Thomas, 2013). In line with the same notion, murine infection models have also shown to be effective in examining DENV pathogenesis and evaluation of vaccine candidates and antiviral drugs (Sarathy et al., 2015).

#### Introducing balance in immunity and reactogenicity

The two way relationship between immunity and reactigenicity has long been discussed with regards to DENV infection. It has been noticed that elevated reactogenicity may lead to a better immune response in some vaccine candidates; nonetheless, severe outcomes may be caused in others. Similarly, lower reactogenicity may result in deficient immune response (Perng et al., 2011). An ideal CYD-TVD vaccine for DENV should be able to minimize the harmful effects along with providing host responses that enhance immune protection and immune evasion. To maintain the proper balance between reactogenicity and immunity, the vital components in the CYD-TDV vaccine play an important role (Perng et al., 2011; Kirkpatrick et al., 2015), which remains a crucial yet biggest task to the vaccine development strategies.

#### Mitigation of the risk of autoimmunity

During current vaccine development strategies, the role of cross-reactive antibodies as mediators of DENV infection has not been the center of attention among the vaccine developers (Nikin-Beers and Ciupe, 2015). Thus, the cross-reactivity of these antibodies has not been considered as a part of the efficacy evaluation index in the clinical trials of CYD-TDV vaccines. However, the details of the protein sequencing in viral antigens eliciting autoimmunity have been well-documented (Perng et al., 2011). Moreover, the significant side effects can be lessened and safety profile of dengue vaccines can be enhanced by applying a strategy, which requires modification of viral genomes genetic code sequence and alteration of these determinants in these altered viral strains (Cheng et al., 2009; Perng et al., 2011).

#### Enhancement of the efficacy of antibody-producing plasma cells

During vaccine development program, the significance of antibody-producing B cells is highly observed. Of the main strategies proposed to improve vaccine potential is the high survivability of plasma or memory B cells (Nothelfer et al., 2015). Furthermore, it has been determined that cysteine-rich interdomain region 1α (CIDR1α) of the *P. falciparum* can defend and save plasma cells from death. Hence, integrating CIDR1α as an additional component with live and/or attenuated dengue vaccines can improve the survivability and functional potential of the plasma cells (Perng et al., 2011).

#### Synthetic nucleic acid antibody immunotherapy

Since none of the current vaccines could provide a balanced protection against DENV, Flingai et al., reported that production of single intramuscular engineered DNA plasmids with human antiviral neutralizing antibodies (nAbs) protected murine models against antibody-modified DENV (Flingai et al., 2015). While the currently used vaccines produce traditional antibodies, the authors emphasized that plasmid-encoded LALA antibodies that defend against DENV and antibody-dependent enhancement (ADE)-induced disease can act as an alternative or become an incentive for traditional vaccine strategies. In fact, this synthetic nucleic acid immunotherapy can also be utilized for traveling population to increase protection against viral infections and reduce the dengue epidemic (Flingai et al., 2015).

### Challenges and limitations to dengue prevention strategies

Just as new strategies and vaccines are devised for prevention and control of DENV, there is always a gap left in the form of challenges and limitations for perfect implementation of such strategies (Achee et al., 2015). Since prevention and control strategies to counter dengue have not shown satisfactory results in reducing disease transmission, the utilization of vaccines as cost-effective and potential resistance has become the main priority to restore public health. However, the complicated immunopathology of dengue has perplexed the development of vaccines. These vaccines also confront various challenges, such as unavailability of suitable models for disease and the want for eligible markers of immunity protection (Ghosh and Dar, 2015).

## Conclusion

As the pandemic outbreak of DENV continues to prevail in today's world, the development of safe, cost-effective, and potential preventive and control measures, including development of new and improved vaccines, evidently promise the reduction of dengue viral infection. As the strategies grow and are used in an integrated manner with other methods, advanced combinations have also predicted attenuation of vector population. Among the vaccines developed, the approbation of recombinant, live and attenuated tetravalent dengue vaccine has proved safe and tolerable, as well as protective against dengue. With more research and experimentation of novel methods and techniques, the future could enjoy better control with protective immunity to DENV.

## Author contributions

IR wrote the paper and designed figure; HP, JBL, and WP. collected the literature; JL, VB, and YP. designed, analyzed approved the paper.

### Conflict of interest statement

The authors declare that the research was conducted in the absence of any commercial or financial relationships that could be construed as a potential conflict of interest.
